# Notes and Comments

**Published:** 1924-08

**Authors:** 


### NOTES AND COMMENTS.

### HOSPITALS AND THE ENTERTAINMENTS DUTY.

THE needs of the hospitals lay at the root of one of the recent defeats of the Government. In Committee on the Finance Bill Lieut.-Colonel Howard Bury, in the interests of the hospitals, moved an amendment exempting from duty any entertainment the whole of the profits of which are devoted to philanthropic, charitable and educational purposes—exemption has hitherto been obtained only when the expenses do not exceed 30 per cent. of the receipts. The Chancellor of the Exchequer, unmoved by Colonel Bury's argument that the hospitals were thus losing a good deal of money which would otherwise go to their support, resisted the amendment. It was, however, carried by a large majority, and, at a later stage, Mr. Snowden made a compromise by raising the allowance for expenses to 50 per cent. This is a fair and reasonable reform, though we think the country could have afforded to remit the tax altogether in cases of this kind. Lord Ednam's amendment to relieve bequests to hospitals from legacy and succession duty was less fortunate. The Chancellor of the Exchequer refused to accept it on the remarkable ground that the proposal " really amounted to a State subsidy to the voluntary hospitals." When politicians are short of arguments they sometimes discover some very surprising ones. Thus Mr. Snowden added that " if legacies to hospitals were freed from duty he did not think that they would gain very much, while the State would lose about £200,000 a year." We should have supposed it to be self-evident that what the State lost the hospitals would gain—that is to say, £200,000 a year! But there is really no particular reason why Chancellors of the Exchequer should be stronger on figures than anyone else.

### "AN AGREED HONORARIUM."

WE have moved a step forward in the vexed matter of payments to the medical staffs of voluntary hospitals. The Representative Body of the British Medical Association, meeting at Bradford, have held further discussions on the subject which once more showed how considerable are the differences of opinion both among medical men and hospital boards. Now, however, the policy of the Association appears to be clear. A resolution was carried declaring that " where the Board of Management of a voluntary hospital accepts contributions for patients from an Approved Society, insurance company, employer of labour, or mass or periodical payments by employees, members of the visiting medical staffs should receive recognition of their services, either in the form of an agreed honorarium or percentage, such payments to be passed into a fund, and such honorarium or fund might be allocated in any manner which the visiting staff might determine." A similar resolution was passed in relation to private patients. To say, as has been argued, that such a course destroys the voluntary principle is to misapprehend the meaning of that principle. It certainly does not depend upon the non-payment of doctors for work which is often arduous and almost always occupies much time. Moreover, the outlook of the voluntary hospitals and their relations to their patients have been so entirely altered by the events of the last few years that it is no longer possible to stand wholly in the ancient ways. **The whole case is very fairly set out in the article on the subject by Mr. C. E. Stewart Flemming on another page.**

### HEALTH IN THE FACTORY.

ONE of the most useful discussions at the Conference of the Royal Sanitary Institute, which has just been held at Liverpool, dealt with the unsatisfactory character of the Factory Bill now before the House of Commons. The measure has made no progress, and it seems likely that there will be abundant opportunity for making the improvements in which, from the health point of view, it so sorely stands in need. The burden of the Liverpool speakers was that, although the Bill contains valuable sanitary provisions, it actually lessens the very small control which the medical staffs of the local authorities now possess over hygienic conditions in factories and workshops. The Medical Officer of Health is denied access to factories save in closely-defined conditions, and the factory inspectors are not medical men and women. Colonel Fremantle, M.P., who read a paper on the subject, described the existing arrangements as an " infernal muddle," and it is obviously a muddled state of things, which charges the Board of Education with the health of school children and the Home Office with the health of factory workers, while neither of them has any effective *liaison* with the Ministry of Health which should be the supreme co-ordinating authority. The truth is that the present systems have grown up haphazard and that reform is much overdue. The disasters which may easily result from these water-tight compartments were luridly illustrated by an instance narrated by Dr. Wynne, the distinguished Medical Officer of Sheffield, in which a number of cases of enteric fever were traced to insanitary conditions in a colliery. The medical officer had no control over that colliery, yet the local authority had to provide the hospital for the treatment of the cases!

### A PILLAR OF CLOUD.

WHEN the Second Reading of a Bill dealing with Smoke Abatement is moved in the House of Lords so late in the Session as July 16, with the explanation that it is on similar lines to those of measures put forward on two previous occasions, and with a pious hope that it may get passed by general agreement, it does not look as if the Government were very much in earnest on the subject. We can but endorse the sentiments expressed by Lord Newton, who said that one hopeful feature was that the question was at any rate one for which the Government had assumed responsibility and that no Government in future would be able to escape that responsibility. It is, of course, intolerable that so pressing a question should have to be the subject of constant " gingering-up," if the term may be allowed, at the hands of any individual, however eminent. Lord Newton commented on the coolness, not to say impudence, of the Government in exempting their own establishments from the Bill. He also saw something more than a mere coincidence in the fact that the most disturbing influences in politics proceeded from the districts most affected by smoke. In the proposed provision that trades which under previous Bills had enjoyed exemption were now going to enjoy still further exemption he traced the hand of Sheffield, which is one of the smokiest of towns. Lord Newton said he was told that airmen, when they rose from London, could distinguish the smoke of Sheffield in the far distance, and could direct their course to it without the aid of their compass, just as the Israelites, in days of old, were able to proceed by means of a pillar of cloud. We can hardly suppose that the people of Sheffield consciously allow their manufacturers to secure exemption from necessary restrictive provisions. If they do, their cloud be upon their own head.

### WHOLESOME BREAD.

WE notice with satisfaction the increase of public interest in the matter of wholesome bread, and there are now several places in the provinces in which true bread, made from English wheat, can be obtained. It is important enough that we should encourage home-grown wheat, but it is even more important that the making of nourishing bread, whatever the origin of its ingredients, should be encouraged. That excessively white bread, chemically bleached, with all the nourishment milled away, lacks some of the essential qualities of a healthy food is notorious, and it is no doubt true that millers find it to their advantage to provide very white flour, and that bakers find it to theirs to bake it insufficiently. But there is another obstacle in the way of wholesome bread—great numbers of people do not want it. When a contemporary indignantly declares that " it is abominable that working-class families, who live so largely on bread, should be unable to obtain a sound and wholesome loaf at a reasonable price," it merely betrays ignorance of the facts. The working-class are precisely those who most ardently adore white bread and " turn up their noses " at any bread which contains the smallest touch of tint. They are profoundly convinced that the whiter it is the better and more genteel it is. Also it is very doubtful whether, as a fact, they do any longer " live so largely on bread." The superior artisan is now so well paid that he is able to live as well as other people, and we all rejoice that it should be so. But he does not consider that he lives well unless he eats the whitest of bread. We sadly need an Order of Bread Missionaries to the Heathen—a class which comprehends the bulk of the population of this country.

### THE FOOD OF THE INSANE.

BROADLY speaking, the food of the patients in County and Borough Mental Hospitals is unpalatable, unattractive, destitute of variety and, in some cases, actually insufficient. That is a not unfair summary of the report of the Committee appointed two years ago by the Board of Control to inquire into this very important subject. There is " undesirable inequality " in the dietary scales, some being well balanced, while others are " lacking in ingenuity " ; as regards quantities, only a bare majority of diets are correct—the other half are " obviously deficient or possibly excessive." In the twelve institutions providing dietaries of the lowest arithmetical value there is an increased incidence of tuberculosis. The partial reinstatement of butter is desirable, and it would be well that there should be a better supply of fresh, and especially of green, vegetables, milk, eggs, salads and wholemeal bread. It would appear that the cost of the Committee's suggested model dietary would not exceed five shillings per week, a sum which they reasonably regard as " economically justifiable." The present inequalities of cost per week per 100 patients are astonishing—Plymouth spends nearly £34 and Lancaster just over £18. Either the one is too high or the other too low ; the Committee's suggested dietary would work out at £25, which is almost exactly the mean of the two extremes. We hope that the proposals of the Committee will be seriously considered by the authorities of the public mental hospitals. It had long been believed, on good evidence, that the food arrangements of these institutions were unsatisfactory, and this report places the matter beyond doubt. We are only beginning to understand fully the influence of food upon health, mental as well as physical, and it is of real importance that the unhappy inmates of asylums should " enjoy any curative or ameliorative advantages that may reside in varied, appetising and well-cooked meals."

### WORK FOR THE TUBERCULOUS.

IT is twenty-six years since Sir Robert Philip developed in the columns of the *British Medical Journal* his theory that work of a definite purposive character, far from impeding the cure of the tuberculous patient, assisted its completion. The article which we print this month on the Papworth Village Settlement serves is an interesting reminder of what has been accomplished, mainly in pursuance of this theory, but also in recognition of the social and family needs of the patients, under the inspiring control of Dr. Varrier Jones. There comes into our hands at the same time a copy of the paper which was read by Dr. Kay Menzies at the recent Conference of the National Association for the Prevention of Tuberculosis. In this paper Dr. Menzies, after emphasising the distinction between Training Colonies and permanent Village Settlements, draws attention to three vitally important considerations in regard to the question of the further extension of Settlements. The ex-service man, by reason of his disability pension and the provision made for his dependants, is in an unusually favourable position for taking full advantage of life in a settlement where he cannot earn a full economic wage ; a large proportion of tuberculosis patients are temperamentally unsuitable for life in a settlement ; and success depends very largely on the personality, inspiration and power of organisation of those in charge. Subject to these three very substantial limitations, it is clear that public and voluntary funds for the prevention and treatment of tuberculosis could not be more happily applied than in the further development of these beneficent institutions. There is another practically untried field in which some good spade work is waiting to be accomplished. The Minister of Health glanced at it in his address at the Conference to which we have referred. It is that of providing under sheltered conditions work for tuberculous patients in the neighbourhood of their own homes, and, as far as possible, at their own trades. It is admittedly a difficult question, but one by no means incapable of settlement. The co-operation of the Trade Unions is essential, and we cannot believe that it will not be forthcoming. Mr. Wheatley can do valuable work in this new field if he has the will.

### CANCER AND DIET.

THE relation of cancer to diet is so much oftener discussed in the nebulous light of sweeping generalities than by the more accurate measure of statistics, that we gladly welcome the latter, particularly when they are handled by so ingenious an exponent of this science as Dr. M. Hindhede, of Denmark. In a report recently issued from his laboratory for the study of dietetics, he has drawn attention to the following facts. Man is the only mammal to die of cancer of the stomach, and civilised man is the only representative of the human family to die of cancer in this position. There are exceptions to this generalisation, but they are nothing more. Cancer in other parts of the digestive tract is also rare in uncivilised communities living on a mild vegetable diet, and betel-chewers suffer mainly from cancer of the skin and mouth. Lower down in the digestive tract it is common to find malignant disease among Danes, whereas it is exceedingly rare among the natives of India. Indeed, in the Danish towns about 60 per cent. of all the cancers are found in the digestive tract, the corresponding figures for the natives of India being only 4 per cent. The difference is probably not determined by a meat or vegetarian diet, for in vegetarian Japan, where rice is highly spiced, cancer of the stomach is very common. In Bavaria, heavy beer-drinkers suffer from a cancer mortality double the average, and in England cancer of the lip is fourteen times, and cancer of the tongue seven times more common in men than in women. But the negro woman who smokes a clay pipe is very subject to cancer of the mouth. Dr. Hindhede associates the high death-rate from cancer in Denmark with his countrymen's groaning tables. " There is hardly any country so rich in fat men and women as Denmark." This form of national wealth is, in his opinion, an asset of more than doubtful value, and he correlates the decline observed during the past ten years in the cancer death-rate with the movement in favour of the simple life. Among Danes over the age of forty-five, every fifth person dies of cancer, an appallingly high risk. But is it in itself likely to induce the man in the street to emulate the dietetic exploits of Nebuchadnezzar ?

### REGISTRATION OF NURSING HOMES.

THERE is much exaggerated talk about the evils existing in nursing homes, but it cannot be denied that there are still a certain number in which the person in charge is untrained and the " nurses " better qualified to wash the floor than to tend the sick. We are therefore glad to learn that the College of Nursing has drafted a Bill " to provide for the Registration and Inspection of Nursing Homes by County Councils and County Borough Councils." The aim has naturally been to make the Bill as little controversial as possible, and, in order that no attendant in a nursing home shall at once be deprived of a living, it provides that only after 1930 must all nurses in these homes be State registered under pain of a fine. The fee to be paid on registration is not to exceed 10s., and a right of appeal is allowed, while a notice must be displayed stating that the home is duly registered. We understand that a clause was first inserted to the effect that nursing homes conducted by medical men should be exempt from inspection. This has, we think rightly, now been omitted. It is obvious that doctors will welcome inspection as much as any other owners of properly conducted and equipped homes, particularly if the inspection be made in their cases by a Medical Officer of Health and not, as alternatively provided by the Bill, by a registered nurse. It is only the bad homes which will oppose inspection. Should the Bill become law it will undoubtedly set at rest many groundless fears as to the way people are treated in these homes, and it will strengthen the position of the owners who are in the main high-principled and public-spirited persons.

### EXERCISE AND VITAMINS.

EVERYONE now knows that vitamins are essential to bodily health, and, if we are to believe advertisements, they are all necessary and in great abundance. But there is no doubt that different conditions of life make different demands on this, as in most other respects, and in the *American Journal of Physiology*, Drs. Kieth and Mitchell report careful researches on this matter. It is unnecessary to detail the exact experiments ; suffice it to say that small animals were given carefully graduated exercise and the best diet determined to produce maximum growth and fitness in each case. It is concluded that active exercise markedly increases our need for the " A " vitamin, whereas no clear evidence could be obtained that any amount or type of muscular work produced any effect on the bodily requirements for vitamin " B." In short, increased muscular activity calls for more vitamin " A." The point may be useful to remember during the outdoor season.

### "NOT PROVEN."

FIFTY-FOUR doctors have met at the Hospital of St. John and St. Elizabeth at St. John's Wood to examine an alleged Lourdes " miracle." After a three hours' investigation they came to the conclusion that " on the evidence submitted the case is not proven." The patient was a young girl who arrived at Lourdes on May 28, 1923, with the fingers of the right hand tightly contracted into the palm, with fingers immovable, and suppurating wounds thereon. She was examined by three doctors before placing her hand in the bath at the spring. Evidence submitted to the doctors gathered at St. John's Wood included a statement by a nurse to the effect that when the girl came out of the bath there was no pus. In the afternoon she had her second bath, and in the evening there was no sign of pus and the wounds were closed. It was a courageous act to assemble so formidable a cohort of medical men, and we may fairly take it as an indication that those who are convinced that the Lourdes waters do accomplish wonders are yet content to submit their belief to scientific tests. It would be just as unscientific to deny these alleged cures without inquiry as to accept them without evidence, and we give due weight to the qualifying words, "on the evidence submitted." We must not talk lightly of "miracles," and, so far as we are aware, there has as yet been no cure at Lourdes of serious organic disease which would have satisfied such a scrutiny as that which has been applied at the Hospital of St. John and St. Elizabeth. In the interests of science we may hope the other cases will receive equally careful investigation.

### CLARIFYING PSYCHO-ANALYSIS.

BOTH within and without the medical profession there is much misunderstanding concerning psycho-analysis. If more simple explanation of the facts were provided, as is done by Dr. Nichols in the *Long Island Medical Journal*, far more use of such methods might be made. The author explains that, for anyone suffering from a functional nervous disorder, we should always be able to do one of two things—either ourselves use intelligent constructive suggestion or refer the patient to a medical expert in these matters. The patient, though his disease is functional—imaginary if you will—is nevertheless definitely in need of treatment. But it is quite unnecessary to follow or even to believe in Freudian concepts and it is unreasonable to lay the blame for all psychological ills to the influence of sex. If instinct is responsible, it need not be the sex instinct. Above all, the technique of psycho-analytical measures can be made reasonably simple. The individual has normally little control over his ideas, but his attention, and therefore his ideas, can be readily directed by a competent adviser. A few aids and peculiarities of method are essential in obtaining complete attention, but that is all. There need be no mystery. The patient tells the physician the disturbing factors of his or her life. The latter then endeavours to develop in his patient the ideas essential to counteract or modify it.

### TRUE AND FALSE VINEGAR.

A BENEVOLENT member of Parliament has brought in a Bill to prevent the sale of imitation vinegar. It has not got beyond its First Reading, and it is only too likely to be numbered among the innocent little measures annually interred beneath the grated floor of the House of Commons. This is a pity, since imitation vinegar made by diluting commercial acetic acid, stained and fermented and flavoured by sulphuric acid, is clearly bad for the human organism. Real malt vinegar is made from alcohol, and has its reasonable domestic and culinary uses. But vinegar, genuine or imitation, has been given uses that are not always reasonable. It is the favourite condiment of the female domestic servant. She eats it with her bacon and her fish, and although she has not actually been detected in putting it into her tea, most housewives, judging by the amazing quantities consumed in the kitchen, are convinced that she thus flavours her preferred beverage. But let us be just. This love of vinegar may not, after all, be merely a vicious taste like the love of soft bread and the detestation of crusts which likewise characterise the neat-handed Phyllis. We have heard it suggested that a generous consumption of vinegar has a whitening effect upon the skin and is conducive to the production of an interesting pallor. But there are external methods, not unfamiliar to her sex, by which this result, supposed to be so alluring, may be produced. Milk and roses, the typical dairymaid complexion, are just now unfashionable. They are too often the product of rude Boreas visiting cheeks too roughly for the happiness of the delicate parlourmaid or even the more robust cook. Nature's own complexions have become as indecorous as the exhibition of ears, but if artificial ones are to be produced by vinegar it is better that it should be the good vinegar which Mr. Hannon, M.P., desiderates.

### OLD BRISBANE HOSPITAL.

AN interesting note of the origin of the Brisbane Hospital is given by Dr. E. Sandford Jackson in the *Medical Journal of Australia*. The medical establishment which came into Moreton Bay with Oxley in 1823 participated, two years later, in the general move from Redcliffe to the present site of Brisbane. At about the beginning of 1825 it seems certain that the Brisbane Hospital was begun at the place where now stands the Supreme Court in George Street. A start was probably made under canvas, and as time went on buildings of bricks and mortar rapidly appeared until, within a few years, there grew on this site a big hospital overlooking the river. As with most early hospitals, the authorities were very hard put to it to find accommodation for all those who needed it, and the earliest annual records, about the year 1827, show some interesting facts. Apparently the total stock of towels in the hospital was fourteen, and it is therefore not surprising that a considerable number of cases were labelled "Ophthalmia!" Erysipelas seems to have been common, but not of very high mortality. The number of cases of rheumatism was proportionately very large, and the disease must have been even more common in those early days than now. Possibly rigorous life and neglect of dental deficiencies both played their part. The number of deaths from dysentery suggests a primitive water supply. But it is noteworthy that with a total admission of 1,050 patients there were in those days only two cases of syphilis and only one of tetanus. In the records, under the heading of *flagellatio*, fifty-one cases are recorded; one patient died. It is not to be supposed that these cases were scattered generally throughout the year, since sometimes a dozen men were flogged in one day. It seems clear that prior to the date under consideration instructions had been given that every man who was flogged should be thereafter admitted to the hospital for such medical treatment as might be necessary. In 1832 there were only two cases of flogging—a pleasant contrast to 1827!

